# Development and expansion of a pediatric transitional pain service to prevent complex chronic pain

**DOI:** 10.3389/fpain.2023.1173675

**Published:** 2023-11-02

**Authors:** Lisa Isaac, Brittany N. Rosenbloom, Jennifer Tyrrell, Danielle A. Ruskin, Kathryn A. Birnie

**Affiliations:** ^1^ Department of Anesthesia and Pain Medicine, The Hospital for Sick Children, Toronto, ON, Canada; ^2^ Department of Anesthesiology and Pain Medicine, Temerty Faculty of Medicine, University of Toronto, Toronto, ON, Canada; ^3^ Child Health Evaluative Sciences, The Hospital for Sick Children, Toronto, ON, Canada; ^4^ Lawrence S. Bloomberg Faculty of Nursing, University of Toronto, Toronto, ON, Canada; ^5^ Department of Psychology, The Hospital for Sick Children, Toronto, ON, Canada; ^6^ Department of Anesthesiology, Perioperative and Pain Medicine, University of Calgary, Calgary, AB, Canada; ^7^ Alberta Children’s Hospital Research Institute, Calgary, AB, Canada

**Keywords:** chronic postsurgical pain, transitional pain service, pediatrics, persistent pain, chronic pain prevention, opioid stewardship

## Abstract

The prevention of chronic pain is a key priority in North America and around the world. A novel pediatric Transitional Pain Service (pTPS) at the Hospital for Sick Children was established to address four main areas of need, which the authors will describe in more detail: (1) provide comprehensive multi-modal pain management and prevention techniques to children at-risk for the development of chronic pain, (2) provide opioid stewardship for children at-risk for chronic pain and their families at home after discharge, (3) facilitate continuity of pain care for children across transitions between inpatient and outpatient care settings, and (4) support caregivers to manage their child's pain at home. The pTPS works with healthcare providers, patients, and their families to address these areas of need and improve quality of life. Furthermore the service fills the gap between inpatient acute pain services and outpatient chronic pain services (accessible only once pain has persisted for >3 months). In pediatric patients who experience pain in hospital and who have been prescribed opioids, discharge to home or rehabilitation may represent a vulnerable time in which pain may persist and during which analgesic requirements may change. This offers an important opportunity to address and prevent the development of chronic pain, and to monitor opioids while ensuring alternative pain therapy is available. The authors will outline risk factors for persistent postsurgical pain, the development and implementation of a pTPS, present initial clinical outcomes andsuggest areas for future research in this evolving area of care.

## Introduction

Preventing chronic pain is a Canadian priority for improving pain care (1). Approximately 20% of children undergoing surgery will develop chronic pain (2). In Canada, this is a large number of children requiring pain care with approximately 80,000 Canadian children undergoing surgery each year (3). Undertreatment of pediatric postoperative pain is widespread, and can delay return to function, lead to long-term opioid use, and negatively impact health-related quality of life, including sleep, anxiety, social and school functioning (4–6). Risk factors for chronic postsurgical pain (CPSP) include child and parent psychological vulnerabilities, pre-operative child pain and disability, postoperative opioid consumption and acute pain (2, 7). Despite known factors to identify children at-risk for CPSP, there remains a dearth of services addressing perioperative care optimization to reduce the development of chronic pain in children (8).

“Transitional Pain Services” are an innovative and effective health service model in adult tertiary care to prevent the transition from acute to chronic pain and oversee opioid stewardship in perioperative care (9). Transitional pain services fill the gap between inpatient acute pain services and outpatient chronic pain services (accessible only once pain has persisted for >3 months). In pediatric patients there is a particularly vulnerable time where patients experience pain in hospital, are prescribed opioids, and then discharged to home or rehabilitation. This timeframe can include a period of prolonged postoperative pain and co-occurring mental health concerns; however, not yet meeting criteria for referral for chronic pain management. Further, analgesic requirements may change, and pain in hospitalized children is undertreated compared to adults, which contribute to poorer functional outcomes (10). Opioid stewardship is essential in pediatric pain care, as nonmedical opioid use in adolescents is often precipitated by exposure through prescription for acute pain management (11). While existing adult transitional pain models offer guidance (9), the need for developmentally-tailored, family-centred pain services for pediatric patients, or a pediatric transitional pain service (pTPS), is recognized (12).

### Risk factors for the development of chronic pain in children

The development of a pTPS requires targets for intervention. As with adults undergoing surgery (13), there are several pediatric risk factors that, if addressed, may alter the transition from acute to chronic pain. Specific child factors that increase the risk of persistent pain include child anxiety (14), depression, fear of pain, pre-existing and immediate postoperative pain (5, 15–17), presurgical functional disability (18), and pain catastrophizing (5, 7, 15, 18, 19). Those with pre-existing pain or pain medication use (20) may also be at increased risk for CPSP. Conversely, pain self-efficacy, or the confidence in one's ability to manage pain, improves pain trajectories in patients with adolescent idiopathic scoliosis (14), and is associated with improved quality of life in adolescents with persistent pain (21). Unique to pediatrics is the influence that caregivers have on their child's pain. For example, parent catastrophic thinking about their child's pain (6, 14), and parent anxiety sensitivity (15) have been shown to be involved in the development of CPSP. Importantly, preoperative expectation management has been found to influence postoperative pain outcomes (i.e., pain control, opioid use) (22, 23). Other currently non-modifiable risk factors for chronic pain include sex, genetics, and epigenetics (24, 25). Risk factors for the transition from acute to chronic pain have been conceptualized through the multifactorial interpersonal fear-avoidance model of chronic pain, a multifactorial model that addresses intersecting developmentally appropriate factors in a child's pain trajectory (26). A pTPS is ideally suited to address these risk factors through a combination of education, psychological intervention, opioid stewardship, medication management, and physical therapy.

### Development and implementation of the pTPS

A novel pTPS was established in 2011 at The Hospital for Sick Children in Toronto, Canada out of a need to fill the gap between acute and chronic pain services. To our knowledge, this was the first pTPS program in the world. The four main goals of the pTPS are to: (1) provide comprehensive multi-modal pain management to children at-risk for the development of chronic pain, (2) provide opioid stewardship for children at-risk for chronic pain and their families at home after discharge, (3) facilitate continuity of pain care for children across transitions between inpatient and outpatient care settings, and (4) support caregivers to manage their child's pain at home. A brief video about the pTPS is available^1^.

Implementation of the pTPS involved ensuring hospital leadership support for, and accessibility of the service. The pTPS began as an *ad hoc* service in a large quaternary pediatric hospital, the Hospital for Sick Children, Toronto, Canada. The model of the pTPS includes initial consultations carried out with pediatric patients and caregivers by an anesthesiologist and advanced practice nurse. Referral for other pTPS-specialist services (i.e., psychologist, physical therapist, and/or occupational therapist with expertise in pediatric complex pain) are provided as needed. The pTPS began as a service consisting of 0.2 + full time equivalent (FTE) anesthesiologist with 0.2 FTE clinical nurse specialist, 0.1 FTE each of patient information coordinator and data analyst, and then 0.2 FTE each of pain physiotherapist and psychologist using existing funding. New funding was secured allowing 1.0 FTE nurse practitioner, 0.4 FTE psychologist, 0.4 FTE physical therapist, and 0.2 FTE each of an occupational therapist, business manager, patient information coordinator and data analyst.

Medical or surgical teams refer to the pTPS when they, their patients or their caregivers may benefit from specialist acute pain management at home or development of an individualized perioperative pain management plan. Referral criteria are based on concerns about perioperative pain management and evidence for risk factors for CPSP. Examples of referrals include: prior pain management challenges; child or parent psychosocial risk factors as discussed above; planned complex surgical interventions, such as scoliosis, Nuss, and congenital foot surgeries, which are known to be painful surgeries; and/or patients who are taking opioids and/or other controlled substances prior to surgery, or for whom weaning is required. Patients with pain for greater than 3 months prior to surgery are seen in the pTPS for presurgical pain management planning, but otherwise are referred to the chronic pain service. Some referrals arise following surgery when complex pain management is required.

Accepted patients are stratified according to risk for CPSP, using Patient Reported Outcomes Measurement Information System (PROMIS)® parent-report and/or developmentally appropriate child and adolescent self-report questionnaires, and additional pTPS-specific questions added (27). These questionnaires are delivered prior to the first visit and intended to identify child (19) and/or parental catastrophizing (3), child and/or parent anxiety, low physical functioning and non-medical substance use vulnerability [using the CRAFFT screening tool (28)], and then in the days and weeks after surgery. These are stratified, according to pTPS patient preferences using the STOP Pain questionnaire (27), such that, for example, questions pertinent to walking are given only to those expected to weight-bear. Parent report is helpful for understanding the health of developmentally-challenged children (29) but also for postoperative patients who may be less able to participate as actively in their care. Pediatric pain questionnaires [PROMIS® pain intensity (29), sleep v.1.0 (30, 31), anxiety v2.0 and depression v2.0 (32) and their parent proxies v2.0 (33), catastrophizing for the child and the parent] are used. The pTPS physician and/or nurse meets with families in hospital or outpatient clinics (in-person and virtually) for a detailed interview at the first visit.

Follow up appointments occur preoperatively where allied health assessments are indicated, and within 7 days of discharge and in the following days and weeks as required. A vulnerable point in the patient trajectory, high pain levels in the weeks after surgery are associated with higher pain levels one year after surgery (15), and close pain follow-up after surgery offers the opportunity to address this. In contrast to adult Transitional Pain Services, pTPS are tailored to the family, recognizing the critical role that families play in child development and in their perioperative care.

**Figure 1:**
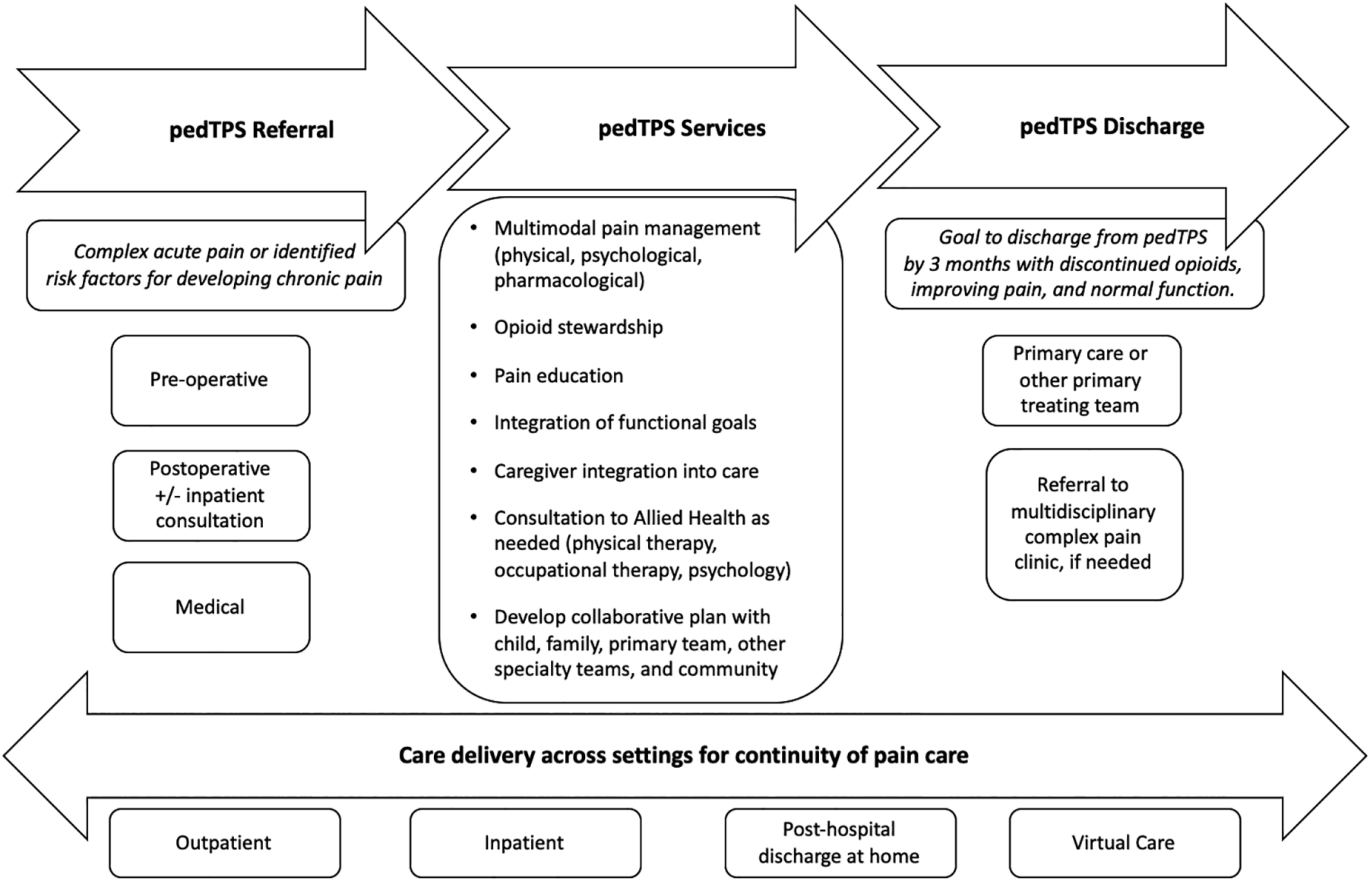
An overview of pediatric transitional pain services at the hospital for sick children.

The pTPS encompasses developmentally-appropriate pediatric pain care throughout the pain continuum, beginning prior to expected painful surgeries, or in medical settings where specialty pain management may be needed at home. Critical to the success of the pTPS, the service coordinates care throughout this entire period and works with families, the surgical teams and anesthesiologists, acute pain services, and other medical/surgical subspecialists (see Figure 1: a schematic of the role of the pTPS in the continuity of pain care).

### Addressing risk factors for chronic post-surgical pain and disability

Confidence in the ability to control pain improves postoperative pain management (14). Addressing this risk factor starts during the initial consultation, where, using a checklist in the form of standardized documentation, education is provided to patients and families about pharmacological and non-pharmacological pain management methods, acute post-operative pain expectations, safe medication administration, storage, monitoring, and disposal. This is reinforced by identifying, recommending, and encouraging appropriate techniques such as online mind-body apps and websites, positioning, safe movement and heat/cold strategies.

Preoperatively, higher pain interference (34) and lower physical functioning (20, 35) may impede the recovery process. The pTPS assesses current physical functioning and engages patients in increased activity or “prehabilitation” (the process of improving the functional capability prior to a surgical procedure, to minimize the effects of postoperative inactivity and its associated decline), if patients have reduced activities of daily living. An umbrella review concludes that there is low certainty evidence that prehabilitation may improve postoperative outcomes (36). Nevertheless, low postoperative activity is associated with a higher incidence of CPSP (20, 37). The pTPS attenuates this by guiding and supporting return to function. For those needing further support for return to normal activities within postoperative surgical guidelines, pTPS physical and occupational therapists are consulted.

To address psychological risk factors, referral to the pTPS psychologist occurs, based on the TPS team's clinical judgement and/or high PROMIS®T scores (greater than 60) for anxiety, depression or catastrophizing. Following an in-depth assessment, the pain psychologist develops individualized psychological pain management and coping strategies and psychological interventions. Education and cognitive behavioral interventions are delivered over 3–4 sessions, approximately weekly prior to surgery. If significant psychological factors are identified from the psychology assessment (e.g., anxiety or mood disorder diagnosis), patients are referred for therapy to address. Acceptance and Commitment Therapy has been shown in studies of adults to help patients to decrease opioid use and pain interference in the perioperative setting (38). For pediatrics, the use of cognitive behavioral therapy, dialectical behavioral therapy, and mindfulness can be helpful. Individualized education addresses psychological contributors to pain perception, the fight or flight system, and gate control theory. Interventions address psychological risk factors (e.g., depression, pain self-efficacy, parental pain catastrophizing) and teach patients and caregivers psychological strategies in preparation for the peri-surgical period, which are reinforced after surgery. For those patients first seen by the pTPS after surgery, the psychologist is consulted to patients who express fear of pain and limitation of progression of functional goals. The pTPS is developing parental strategies in addition to supporting patients individually.

The pTPS allows for optimization of pain management planning and associated psychological vulnerabilities. For example, surgical deferral has allowed time to address psychological vulnerabilities identified during psychological assessment. This provides the time and change required to improve readiness for surgery and to optimize post-surgical outcomes (eg., eating disorders, psychotic disorders). Discharge from pTPS occurs once opioids are discontinued, pain is improving and trending towards baseline, or normal function has resumed, aiming for a maximum follow-up of 3 months. If pain and poor function continues beyond this time, referral to the pediatric chronic pain clinic (or young adult) occurs.

### Opioid stewardship

Opioid stewardship is equally important to perioperative and pain care. Although uncommon, with 10% receiving a repeat prescription in the United States (39), several unique situations occur with opioid prescriptions for youth. Some adolescents may be at higher risk of opioid use disorder, and while detection of risk in adults is informed by prior experience with non-prescribed substances, screening for adolescents relies on vulnerability for any substance use [e.g., the CRAFFT screener (28)] and understanding protective factors. Children and adolescents generally live in a home with adults who may be responsible for distributing medications, which may be protective. Patients frequently live with other children or bring others into the home, who may be susceptible to inadvertent or even purposeful ingestion or diversion of opioids. Minimization of excess opioids in the home is critical to preventing this. We know that perioperative opioid prescription requirements are extremely variable (40, 41), and that excess opioids are common following prescription (42, 43). The pTPS allows very short opioid prescription for patients at discharge, as patients are seen within 3–7 days of discharge, and a primary contact is available in the interim for pain concerns. Non-opioid pharmacological support by the pTPS includes instruction or prescription of acetaminophen and non-steroidal anti-inflammatory medications to optimize response and side effects. In some cases, gabapentinoids are prescribed, titrating doses pre-operatively to minimize side effects, and contributing to optimal pain management, such as prior to minimally-invasive pectus excavatum surgeries (44). Muscle relaxants such as methocarbamol and/or low dose diazepam are weaned by the pTPS following some surgeries, and generally completed within two weeks of discharge from hospital. Current recommendations are to limit discharge opioid prescriptions, using the lowest effective dose for the shortest duration possible (45). Postoperative opioid requirements varied with the type of surgery in veterans, suggesting that optimal management includes close follow up and optimization of non-pharmacological methods of pain control (46). While greater opioid doses can increase the risk of opioid overdose (47), access to opioids in the home also increases this risk (48). In a nonrandomized trial of adult transitional pain services, opioid reduction with improvements in pain management is realized with the use of the Transitional Pain Service (9, 38). In the pediatric setting, the pTPS screens for those at risk for prolonged pain and potential opioid use and opioid use disorder. The pTPS manages and monitors opioid prescriptions while offering alternative pain management strategies.

### Demographics of the pTPS

**Figure 2:**
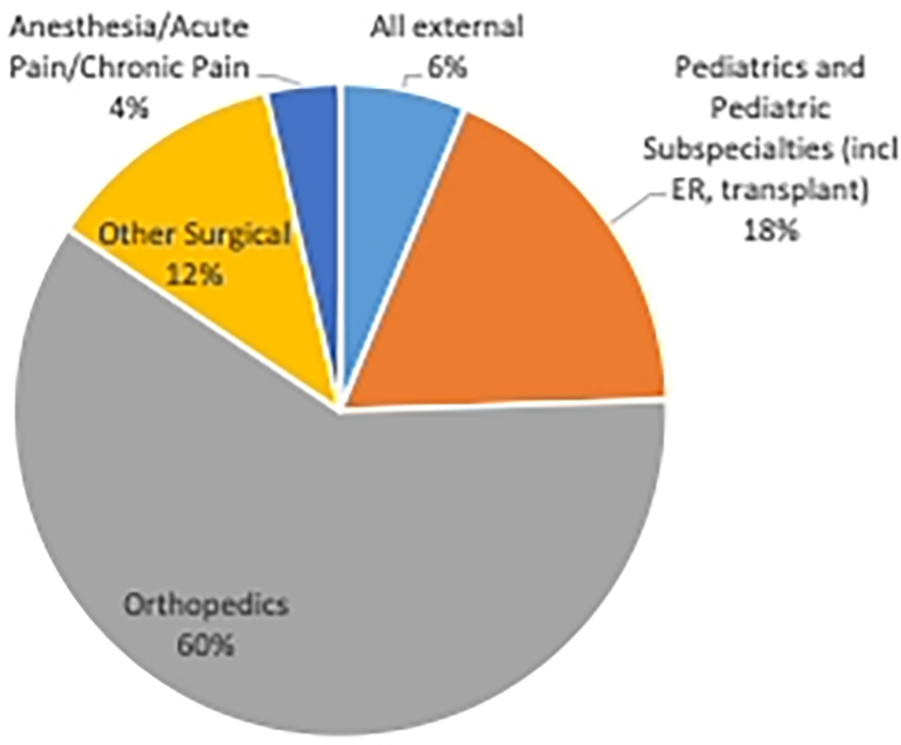
Referrals to the pediatric transitional pain service 2021-2022.

By the 2015–2016 fiscal year, pTPS assessed 10 children and adolescents for the first time. Referrals have increased substantially since inception. In the 2019–2020 fiscal year the pTPS provided service to 101 children and adolescents from 4 to 21 years of age (56% female). The Covid-19 pandemic impacted referral to the service due to Canadian hospital restrictions of surgeries as at least 72% of patients seen are surgical patients (e.g., Orthopedics, general surgery). Another 4%, primarily surgical patients, are referred from acute or chronic pain or pre-anesthesia services. Referral patterns increased following initial Covid-19 operating room shutdowns, with the pTPS seeing 156 new patients in 2021–2022 fiscal year. A portion of patients are referred to the pTPS from medical specialties (e.g., oncology, complex pediatric care, emergency medicine) (see Figure 2). Most children required an average of 2.75 visits, with outliers requiring as many as 10 visits due to case complexity.

As part of an enhanced recovery after surgery protocol, the pTPS has contributed to reduction of hospital length of stay (44, 49). Early discharge appeared to be challenging for families initially, with patients reporting high pain over the first days at home. According to patient and caregiver feedback, the use of detailed instructions for home, including contact information for the pTPS and regular follow up has resulted in improved pain management in both the first few days and the first few weeks and average length of follow up of 2 or 3 weekly visits for scoliosis and pectus excavatum repair. Hospital lengths of stay were 1–2 days for pectus excavatum and 2–3 days following scoliosis repair. In the 2021–2022 fiscal year, 96% of respondents completing our satisfaction survey felt the support they received from the pTPS was good to excellent.

Approximately 30% of all new patients are referred to a pTPS pain psychologist by the pTPS anesthesiologist, as identified by clinical assessment or questionnaires as described above. A portion (7.8%) of patients went on to be seen in the chronic pain clinic for ongoing interdisciplinary pain care in the 2019–2020 cohort, and only 2.6% in 2021–2022. This compares favorably to the expected incidence of CPSP in most studies of 20% (3).

## Discussion

Ideally positioned as a perioperative care link, and geared towards patient-reported outcomes, the pTPS uses both parent proxy and patient reported data to guide therapy and integrate pain care. Particularly in the pediatric setting, including family members in the development of a perioperative pain management plan to help with understanding recovery and difficulties with therapy can improve management.

Children seen by the pTPS have greater access to pain treatment, including non-opioid medication, psychological consultation, and mind-body therapies along with physical and occupational therapies preceding and following surgery. The service offers close opioid monitoring, appropriate tapering and emphasizes psychological and physical pain management strategies through its adherence to a biopsychosocial model of pain treatment. Using perioperative pain management expertise, the service allows discharging services to adhere to recommendations for limited opioid prescribing (50), while ensuring appropriate follow up and the ability to match opioid requirements to prescriptions.

Patients with chronic pain cost the Ontario healthcare system 50% more than those without chronic pain, an incremental cost of $1,742 CDN in 2014 (51), or $2,191.01 today. Prevention of chronic pain should reduce some of these costs. But robust evidence for this is lacking. Costs of running a new service are primarily related to personnel costs (52), offset by a reduction, for example, of $10,490 per hospital day saved (53) by close pain follow-up. Although the pTPS has seen significant increases in patient numbers since its inception, the proportion of patients seen by the chronic pain clinic following pTPS is well below the established incidence of pediatric post-surgical chronic pain in the literature of approximately 20% (3), and specifically 22% at our institution (54). Offering virtual visits also results in cost savings for families, and reduces the impact of parental leave from work.

The pediatric CPSP literature does not address which surgeries increase risk (2), and referral patterns to our service may be unique. However, adults having bone surgery appear to be at greater risk for chronic pain (55), and most patients referred to the pTPS undergo orthopedic surgery.

As our understanding of perioperative outcomes expands, multiple areas exist for further research which benefit from an established Transitional Pain Service. We know that patients and their caregivers are interested in perioperative pain management support, and that healthcare providers would like evidence-based psychological therapies, prehabilitation, preventive medications and new therapies in pediatric settings to minimize the chance of persistent postsurgical pain (56). Referrers are often in busy surgical clinics, and a validated, rapid checklist or screening measure would facilitate their recognition of potential candidates for pTPS, so that patients and their families can benefit from pain education and the development of a pain management plan and support. Outcomes such as opioid dosing and duration, and functional outcomes such as time to return to school, baseline social engagement or to expected physical activities and long-term consequences of their delay must be evaluated. Definitive evidence of the efficacy of specific psychological therapies, such as cognitive behavioral therapy or acceptance and commitment therapy and of the pTPS to prevent or minimize the development of chronic postsurgical pain in children, is needed.

The pTPS is uniquely situated to recognize children at risk for developing chronic pain and to prevent its development, while encouraging comprehensive pain management. Thus, a pTPS would best be delivered by: (1) becoming a full capacity interdisciplinary team including a pain psychologist, physiotherapist, and occupational therapist to address all potentially modifiable risk factors; (2) expanded access to remote, rural, and underserviced areas and exposure of children to specialty pain care; and (3) integration within all care pathways for children undergoing major surgery and those vulnerable to the development of chronic pain (e.g., complex care). As the first pTPS internationally to our knowledge, we are poised to conduct research, assessing factors that alter the postoperative course, and identify areas where intervention may prevent the development of chronic pain.

## Data Availability

The raw data supporting the conclusions of this article will be made available by the authors, without undue reservation.

## Footnotes

^1^ https://youtu.be/OJdXrMX8nWg

## References

[1] Canadian Pain Task Force. *Chronic Pain in Canada: Laying a Foundation for Action*. Government of Canada; 2019 Jun. Available at: https://www.canada.ca/en/health-canada/corporate/about-health-canada/public-engagement/external-advisory-bodies/canadian-pain-task-force/report-2019.html#pre

[2] Rabbitts JA Fisher E Rosenbloom BN Palermo TM. Prevalence and predictors of chronic postsurgical pain in children: a systematic review and meta-analysis. J Pain. (2017) 18(6):605–14. 10.1016/j.jpain.2017.03.007 28363861 PMC5457338

[3] Wright JG Menaker RJ, the Canadian Paediatric Surgical Wait Times Study Group. Waiting for children’s surgery in Canada: the Canadian paediatric surgical wait times project. Can Med Assoc J. (2011) 183(9):E559–64. 10.1503/cmaj.101530 21543299 PMC3114932

[4] Sieberg CB Simons LE Edelstein MR DeAngelis MR Pielech M Sethna N Pain prevalence and trajectories following pediatric spinal fusion surgery. J Pain. (2013) 14(12):1694–702. 10.1016/j.jpain.2013.09.005 24290449 PMC3873090

[5] Rabbitts JA Zhou C Groenewald CB Durkin L Palermo TM. Trajectories of postsurgical pain in children: risk factors and impact of late pain recovery on long-term health outcomes after major surgery. PAIN. (2015) 156(11):2383–9. 10.1097/j.pain.0000000000000281 26381701 PMC4607609

[6] Harbaugh CM Lee JS Hu HM McCabe SE Voepel-Lewis T Englesbe MJ Persistent opioid use among pediatric patients after surgery. Pediatrics. (2018) 141(1):e20172439. 10.1542/peds.2017-2439 29203521 PMC7053700

[7] Rosenbloom BN Pagé MG Campbell F Isaac L Stinson JN Wright JG Pediatric chronic postsurgical pain and functional disability: a prospective study of risk factors up to one-year after major surgery. J Pain Res. (2019) 12:3079–98. 10.2147/JPR.S210594 31814752 PMC6858804

[8] Williams G Howard RF Liossi C. Persistent postsurgical pain in children and young people: prediction, prevention, and management. PAIN Reports. (2017) 2(5):e616. 10.1097/PR9.0000000000000616 29392231 PMC5777679

[9] Katz J Weinrib AZ Clarke H. Chronic postsurgical pain: from risk factor identification to multidisciplinary management at the Toronto general hospital transitional pain service. Can J Pain. (2019) 3(2):49–58. 10.1080/24740527.2019.1574537 35005419 PMC8730596

[10] Friedrichsdorf SJ Goubert L. Pediatric pain treatment and prevention for hospitalized children. Pain Rep. (2019) 5(1):e804. 10.1097/PR9.0000000000000804 32072099 PMC7004501

[11] McCabe SE West BT Boyd CJ. Leftover prescription opioids and nonmedical use among high school seniors: a multi-cohort national study. J Adolesc Health. (2013) 52(4):480–5. 10.1016/j.jadohealth.2012.08.007 23298996 PMC3608842

[12] Birnie KA Stinson J Isaac L Tyrrell J Campbell F Jordan IP Mapping the current state of pediatric surgical pain care across Canada and assessing readiness for change. Can J Pain. (2022) 6(2):108–20. 10.1080/24740527.2022.2038031 35692556 PMC9176261

[13] Katz J Seltzer Z. Transition from acute to chronic postsurgical pain: risk factors and protective factors. Expert Rev Neurother. (2009) 9(5):723–44. 10.1586/ern.09.20 19402781

[14] Connelly M Fulmer RD Prohaska J Anson L Dryer L Thomas V Predictors of postoperative pain trajectories in adolescent idiopathic scoliosis. Spine. (2014) 39(3):E174–181. 10.1097/BRS.0000000000000099 24173016

[15] Pagé MG Campbell F Isaac L Stinson J Katz J. Parental risk factors for the development of pediatric acute and chronic postsurgical pain: a longitudinal study. J Pain Res. (2013) 6:727–41. 10.2147/JPR.S51055 24109194 PMC3792832

[16] Rosenbloom BN Slepian PM Pagé MG Isaac L Campbell F Stinson J Differential risk factor profiles in the prediction of general and pain-specific functional limitations 12 months after major pediatric surgery. Children (Basel). (2021) 8(5):360. 10.3390/children8050360 33946246 PMC8146066

[17] Noel M Rabbitts JA Tai GG Palermo TM. Remembering pain after surgery: a longitudinal examination of the role of pain catastrophizing in children’s and parents’ recall. Pain. (2015) 156(5):800–8. 10.1097/j.pain.0000000000000102 25630028 PMC4402244

[18] Birnie KA Chorney J El-Hawary R Group PS. Child and parent pain catastrophizing and pain from presurgery to 6 weeks postsurgery: examination of cross-sectional and longitudinal actor-partner effects. Pain. (2017) 158(10):1886–92. 10.1097/j.pain.0000000000000976 28598902

[19] Chidambaran V Ding L Moore DL Spruance K Cudilo EM Pilipenko V Predicting the pain continuum after adolescent idiopathic scoliosis surgery: a prospective cohort study. Eur J Pain. (2017) 21(7):1252–65. 10.1002/ejp.1025 28346762 PMC5541247

[20] Batoz H Semjen F Bordes-Demolis M Bénard A Nouette-Gaulain K. Chronic postsurgical pain in children: prevalence and risk factors. A prospective observational study. Br J Anaesth. (2016) 117(4):489–96. 10.1093/bja/aew260 28077537

[21] Grasaas E Helseth S Fegran L Stinson J Småstuen M Haraldstad K. Health-related quality of life in adolescents with persistent pain and the mediating role of self-efficacy: a cross-sectional study. Health Qual Life Outcomes. (2020) 18(1):19. 10.1186/s12955-020-1273-z 32000787 PMC6993393

[22] Rucinski K Cook JL. Effects of preoperative opioid education on postoperative opioid use and pain management in orthopaedics: a systematic review. J Orthop. (2020) 20:154–9. 10.1016/j.jor.2020.01.020 32025140 PMC6997111

[23] Yang D Jha S Swallow J Caird MS Lopyan A Stepanovich M Preoperative patient education and smaller prescription quantity reduce opioid use after posterior spinal fusion for adolescent idiopathic scoliosis: results of a prospective study. J Pediatr Orthop. (2022) 42(8):e868–73. 10.1097/BPO.0000000000002215 35856498

[24] Chidambaran V Zhang X Martin LJ Ding L Weirauch MT Geisler K DNA Methylation at the mu-1 opioid receptor gene (*OPRM1*) promoter predicts preoperative, acute, and chronic postsurgical pain after spine fusion. Pharmgenomics Pers Med. (2017) 10:157–68. 10.2147/PGPM.S132691 28533693 PMC5432115

[25] Chidambaran V Zhang X Geisler K Stubbeman BL Chen X Weirauch MT Enrichment of genomic pathways based on differential DNA methylation associated with chronic postsurgical pain and anxiety in children: a prospective, pilot study. J Pain. (2019) 20(7):771–85. 10.1016/j.jpain.2018.12.008 30639570 PMC6616015

[26] Rosenbloom BN Katz J. Modeling the transition from acute to chronic postsurgical pain in youth: a narrative review of epidemiologic, perioperative, and psychosocial factors. Can J Pain. (2022) 6(2):166–74. 10.1080/24740527.2022.2059754 35711297 PMC9196786

[27] Stratton C Tyrrell J Goren R Lalloo C Isaac L. The “STOP pain” questionnaire: using the plan-do-study-act model to implement a patient-family preferences-informed questionnaire into a pediatric transitional pain clinic. J Patient Rep Outcomes. (2022) 6(1):120. 10.1186/s41687-022-00520-4 36445535 PMC9708994

[28] Knight JR Sherritt L Shrier LA Harris SK Chang G. Validity of the CRAFFT substance abuse screening test among adolescent clinic patients. Arch Pediatr Adolesc Med. (2002) 156(6):607–14. 10.1001/archpedi.156.6.607 12038895

[29] Lifland BE Mangione-Smith R Palermo TM Rabbitts JA. Agreement between parent proxy report and child self-report of pain intensity and health-related quality of life after surgery. Acad Pediatr. (2018) 18(4):376–83. 10.1016/j.acap.2017.12.001 29229566 PMC5936667

[30] Bevans KB Meltzer LJ De La Motte A Kratchman A Viél D Forrest CB. Qualitative development and content validation of the PROMIS pediatric sleep health items. Behav Sleep Med. (2019) 17(5):657–71. 10.1080/15402002.2018.1461102 29693445

[31] Forrest CB Meltzer LJ Marcus CL de la Motte A Kratchman A Buysse DJ Development and validation of the PROMIS pediatric sleep disturbance and sleep-related impairment item banks. Sleep. (2018) 41(6):zsy054. 10.1093/sleep/zsy054 29546286

[32] Quinn H Thissen D Liu Y Magnus B Lai JS Amtmann D Using item response theory to enrich and expand the PROMIS® pediatric self report banks. Health Qual Life Outcomes. (2014) 12:160. 10.1186/s12955-014-0160-x 25344155 PMC4212129

[33] Irwin DE Gross HE Stucky BD Thissen D DeWitt EM Lai JS Development of six PROMIS pediatrics proxy-report item banks. Health Qual Life Outcomes. (2012) 10:22. 10.1186/1477-7525-10-22 22357192 PMC3312870

[34] Giordano NA Kent ML Kroma RB Rojas W Lindl MJ Lujan E Acute postoperative pain impact trajectories and factors contributing to trajectory membership. Pain Med. (2023) 24(7):829–36. 10.1093/pm/pnac203 36579887

[35] Uyama K Ida M Wang X Naito Y Kawaguchi M. Association of preoperative functional disability with chronic postsurgical pain: a prospective observational study. Eur J Pain. (2022) 26(4):902–10. 10.1002/ejp.1918 35104389

[36] McIsaac DI Gill M Boland L Hutton B Branje K Shaw J Prehabilitation knowledge network. Prehabilitation in adult patients undergoing surgery: an umbrella review of systematic reviews. Br J Anaesth. (2022) 128(2):244–57. 10.1016/j.bja.2021.11.014 34922735

[37] Rabbitts JA Holley AL Zhou C Chen L. Physical activity as a predictor of chronic pain following pediatric spinal surgery. Clin J Pain. (2021) 37(3):186–93. 10.1097/AJP.0000000000000903 33273273 PMC7867602

[38] Azam M A Weinrib AZ Montbriand J Burns LC McMillan K Clarke H Acceptance and commitment therapy to manage pain and opioid use after major surgery: preliminary outcomes from the Toronto general hospital transitional pain service. Can J Pain. (2017) 1(1):37–49. 10.1080/24740527.2017.1325317 35005340 PMC8730651

[39] Nair AA Placencia JL Farber HJ Aparasu RR Johnson M Chen H. Association between initial opioid prescription duration and 30-day risk of receiving repeat opioid among children. Acad Pediatr. (2023) 23(2):416–24. 10.1016/j.acap.2022.06.006 35863737

[40] Sun N Steinberg BE Faraoni D Isaac L. Variability in discharge opioid prescribing practices for children: a historical cohort study. Can J Anaesth. (2022) 69(8):1025–32. English. 10.1007/s12630-021-02160-6 34904210

[41] Horton JD Munawar S Corrigan C White D Cina RA. Inconsistent and excessive opioid prescribing after common pediatric surgical operations. J Pediatr Surg. (2019) 54(7):1427–31. 10.1016/j.jpedsurg.2018.07.002 30057208

[42] Caldeira-Kulbakas M Stratton C Roy R Bordman W Mc Donnell C. A prospective observational study of pediatric opioid prescribing at postoperative discharge: how much is actually used? Can J Anaesth. (2020) 67(7):866–76. English. 10.1007/s12630-020-01616-5 32166621

[43] Raina J Costello C Suarthana E Tulandi T. Postoperative discharge opioid consumption, leftover, and disposal after obstetric and gynecologic procedures: a systematic review. J Minim Invasive Gynecol. (2022) 29(7):823–831.e7. 10.1016/j.jmig.2022.04.017 35513302

[44] Downing L Ramjist JK Tyrrell A Tsang M Isaac L Fecteau A. Development of a five point enhanced recovery protocol for pectus excavatum surgery. J Pediatr Surg. (2023) 58(5):822–7. 10.1016/j.jpedsurg.2023.01.028. 36788057

[45] *Choosing Wisely Canada*. Available at: https://choosingwiselycanada.org/opioid-wisely/#recommendation (Accessed February 19, 2023).

[46] Scully RE Schoenfeld AJ Jiang W Lipsitz S Chaudhary MA Learn PA Defining optimal length of opioid pain medication prescription after common surgical procedures. JAMA Surg. (2018) 153(1):37–43. 10.1001/jamasurg.2017.3132 28973092 PMC5833616

[47] Chua KP Brummett CM Conti RM Bohnert A. Association of opioid prescribing patterns with prescription opioid overdose in adolescents and young adults. JAMA Pediatr. (2020) 174(2):141–8. 10.1001/jamapediatrics.2019.4878 31841589 PMC6990690

[48] Nguyen AP Glanz JM Narwaney KJ Binswanger IA. Association of opioids prescribed to family members with opioid overdose among adolescents and young adults. JAMA Netw Open. (2020) 3(3):e201018. 10.1001/jamanetworkopen.2020.1018 32219404 PMC7462253

[49] Lebel DE Machida M Koucheki R Campbell F Bath N Koyle M Utilization of individual components of enhanced recovery after surgery (ERAS) protocol improves post-operative outcomes in adolescent idiopathic scoliosis: a blueprint for progressive adoption of ERAS. Spine Deform. (2023) 11(5):1117–25. 10.1007/s43390-023-00706-w 37233951 PMC10425294

[50] Vetter TR Kain ZN. Role of the perioperative surgical home in optimizing the perioperative use of opioids. Anesth Analg. (2017) 125(5):1653–7. 10.1213/ANE.0000000000002280 28742770

[51] Hogan ME Taddio A Katz J Shah V Krahn M. Incremental health care costs for chronic pain in Ontario, Canada: a population-based matched cohort study of adolescents and adults using administrative data. Pain. (2016) 157(8):1626–33. 10.1097/j.pain.0000000000000561 26989805

[52] Canadian Institute for Health Information. *Hospital spending*. (Accessed August 25, 2023).

[53] Canadian Institute for Health Information. *Cost of a Standard Hospital Stay*. Available at: https://yourhealthsystem.cihi.ca/hsp/endetail?lang=en#/indicator/015/4/O5109/ (Accessed August 25, 2023).

[54] Pagé MG Stinson J Campbell F Isaac L Katz J. Identification of pain-related psychological risk factors for the development and maintenance of pediatric chronic postsurgical pain. J Pain Res. (2013) 6:167–80. 10.2147/JPR.S40846 23503375 PMC3594915

[55] van Driel MEC van Dijk JFM Baart SJ Meissner W Huygen FJPM Rijsdijk M. Development and validation of a multivariable prediction model for early prediction of chronic postsurgical pain in adults: a prospective cohort study. Br J Anaesth. (2022) 129(3):407–15. 10.1016/j.bja.2022.04.030 35732539

[56] Birnie KA Dib K Ouellette C Dib MA Nelson K Pahtayken D Partnering for pain: a priority setting partnership to identify patient-oriented research priorities for pediatric chronic pain in Canada. CMAJ Open. (2019) 7(4):E654–64. 10.9778/cmajo.20190060 31699686 PMC6839970

